# P-1257. Cefazolin Dosage Considerations for Methicillin-Susceptible *Staphylococcus aureus* (MSSA) Central Nervous System Infections: Exploring Optimal Regimens

**DOI:** 10.1093/ofid/ofae631.1439

**Published:** 2025-01-29

**Authors:** C Tyler Pitcock, David S Burgess, Katie B Olney

**Affiliations:** Duke University Health, Durham, North Carolina; University of Kentucky College of Pharmacy, Lexington, Kentucky; University of Kentucky HealthCare, Lexington, Kentucky

## Abstract

**Background:**

Historically, cefazolin (CFZ) has not been considered an appropriate agent to treat central nervous system (CNS) infections. Recent studies have shown that standard CFZ dosing achieves concentrations above the epidemiologic cutoff of minimum inhibitory concentration ≤ 2 mg/L for *Staphylococcus aureus*, but literature describing optimal dosing for CNS infections is lacking. This study aimed to identify CFZ dosing regimens necessary to achieve adequate probability of target attainment (PTA) for treatment of a methicillin-susceptible *S. aureus* (MSSA) CNS infection.

**Figure:**
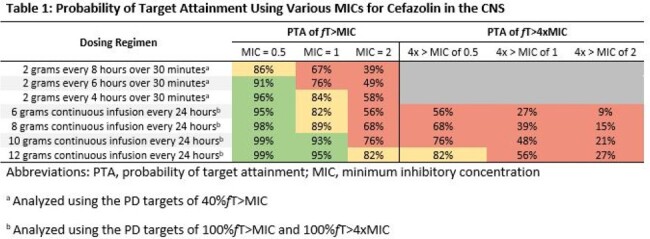

**Methods:**

CFZ plasma and cerebral spinal fluid (CSF) pharmacokinetic parameters were obtained from a previous published study. Monte Carlo simulations were performed utilizing PD targets of 40%*f*T > MIC for intermittent infusions and 100%*f*T > MIC and 100%*f*T > 4xMIC for continuous infusions (CI) to determine probability of target attainment (PTA) for seven CFZ dosing regimens at an MIC of 0.5 mg/L, 1 mg/L, and 2 mg/L. Adequate target attainment, defined as ≥ 90% PTA, was evaluated for CFZ dosing regimens ranging from 6-12g/day administered intermittently every 4-8 hours over 30 minutes or as a CI over 24 hours (**Table 1**).

**Results:**

The standard intermittent regimen of 2g q8h did not achieve ≥ 90% PTA for any MIC evaluated. Furthermore, no CI regimen achieved ≥ 90% PTA when using the PD target of 100%*f*T > 4xMIC. All continuous and intermittent regimens achieved ≥ 90% PTA when the MIC remained ≤ 0.5 mg/L. Only the 10 g and 12 g CI regimens achieved ≥ 90% PTA for 100%*f*T > MIC when the MIC was 1 mg/L while no regimen achieved the desired target when the MIC was 2 mg/L.

**Conclusion:**

These findings underscore the inadequacy of standard intermittent CFZ regimens in achieving desired PD targets for MSSA CNS infections. Higher total daily doses or CI CFZ is necessary to achieve satisfactory PD targets for MSSA CNS infections. When comparing the same total daily dose administered as an intermittent versus continuous infusion, our results suggest that continuous infusions are more likely to achieve PD target attainment for MSSA CNS infections, particularly when the cefazolin MIC exceeds 0.5 mg/L. Further clinical studies are warranted to evaluate the safety and efficacy of CFZ for CNS infections caused by MSSA.

**Disclosures:**

**All Authors**: No reported disclosures

